# Immune-mediated adverse events post-COVID vaccination and types of vaccines: a systematic review and meta-analysis

**DOI:** 10.1186/s43162-022-00129-5

**Published:** 2022-05-19

**Authors:** Hind A. ElSawi, Ahmed Elborollosy

**Affiliations:** 1 Department of Internal Medicine, Badr University in Cairo, Badr City, Egypt; 2 Department of Internal Medicine, Modern University for Technology and Information, Cairo, Egypt

**Keywords:** COVID vaccine, Viral vector-based AstraZeneca, Moderna, Pfizer J&J, Adverse events post-vaccine, Immunological, Thrombosis, Thrombocytopenia, Glomerulonephritis, Neurological risk profile

## Abstract

**Background:**

In late 2019, Coronavirus disease 2019 has been declared as a global emergency by World Health Organization. Hopefully, recent reports of effective and safe vaccines were welcomed, and approved on emergency base. Millions of recipients had received one of the approved COVID 19 vaccines, with lots of adverse events recorded global wide.

**Objective:**

To assess post-COVID vaccination immune-mediated adverse events and evaluate its association to specific type of vaccine global wide.

**Methods:**

Systematic literature review and meta-analysis of published reports (since December 2020 till December 2021) on immune-mediated adverse events post-COVID vaccination.

**Results:**

We evaluated 34 published studies; 460 cases with various adverse events post-COVID vaccination. Studies in current literature are primarily retrospective case series, isolated case reports or narrative studies. Different COVID vaccines were involved. Results’ data was subcategorized according to associated vaccine. Adverse effects of COVID-19 vaccinations included thrombotic, neurological, myocarditis, ocular, dermatological, renal, hematological events timely linked to inoculation. Each vaccine type was linked to adverse profile that differ from others.

**Conclusion:**

High suspicion of post-vaccination adverse events is mandatory to provoke earlier detection, better understanding, optimum prevention, and management. Specific vaccine/patient risk profile is needed to selectively categorize target population to reduce morbidity and mortality post-vaccination.

## Introduction

Coronavirus disease 2019 (COVID-19), has been announced in late 2019 by WHO as global pandemic. It varies from asymptomatic to severe respiratory distress syndrome. Millions of infected people as well as deaths had been reported all over the world. A year later, many vaccines against COVID-19 were announced and approved on international base. Since emergency approvals of COVID vaccines, a number of concerns about their reactogenicity have been raised. This could be translated into a polyclonal B cell expansion, immune complex formation, and vasculitic phenomena [1].

In most vaccinated recipients, vaccine antigens are recognized by immune system with stimulation of local immune cells followed by recruitment of circulating immune cells and then, vasodilators and cytokines trigger local inflammation. So, adequate vaccine reactogenecity is essential for protective responses without substantial systemic effects [2].

Antigenic similarity between the SARS-COV-2 spike protein and human proteins causes anti-SARS-COV2 antibodies to bind to human antigens, such as extractable nuclear antigens, nuclear antigen, and myelin basic proteins. In case of hyper reactogenecity, vasodilators and cytokines enter the bloodstream and induce a systemic inflammatory response syndrome [2].

Among studied vaccines are viral vector laden, mRNA-based and inactivated vaccines.

This review provides a comprehensive overview of COVID-19 vaccine-induced immune adverse effects. Adverse events include thrombotic, renal, cardiac, dermatological, ocular, and hematological events. A review of such conditions is timely and would be beneficial to physicians and healthcare professionals alike, in identifying patients who may be at a higher risk so that protocols for close monitoring can be designed and implemented, as well as risk profiling of vaccines, to configure vaccine target population, and hence reduce morbidity and mortality post-vaccination.

## Methodology

Systematic reviews, a cornerstone of evidence-based medicine (EBM). EBM uses the best available research evidence along with clinical experience and patient needs and expectations [3].

For this systematic review, a literature search was conducted using the databases PubMed and Google Scholar and applying the search terms “SARS-CoV-2 vaccination”, “Covid vaccine”, “mRNA based vaccine”, “vector-based vaccine” “inactivated vaccine” combined with “side effect”, “adverse reaction”, “polyradiculitis”, “neuropathy”, “Miller-Fisher syndrome”, “Guillain-Barre syndrome”, “myocarditis”, “thrombophilia”, “thrombosis”, “glomerulopathy”, “vasculitis”, “thrombocytopenia”, “myopathy “, “erythema nodosum “, dermatomyositis, “Steven Johnson syndrome”, “uveitis”.

**Fig. 1:**
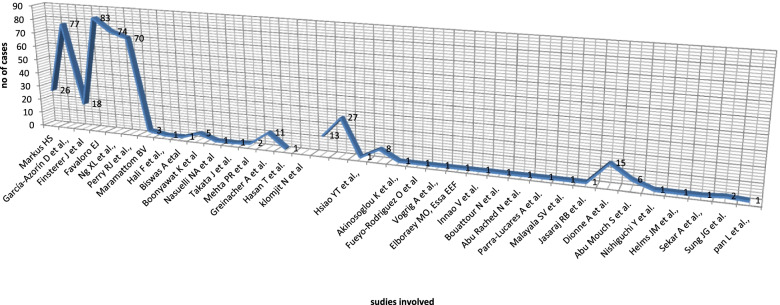
Chart of involved studies by name and number of cases

Additionally, reference lists from the available articles were further checked. Articles that provided detailed information about individual patients experiencing any immune-mediated adverse effect after the first or second dose of the SARS-CoV-2 vaccine were included. We restricted our search to articles published within the past decade, up till December, 2021 (Fig. 1).

### Exclusion criteria

Articles that were not accessible or only available as an abstract or in a language other than German, English, French, or Spanish were excluded. Studies focusing on adverse symptoms rather than adverse diagnosis were excluded. Studies with overlap of published cases were excluded as well.

## Results

The included studies were 34 studies. According to type of vaccine, 21 study of viral vector-based vaccines (Oxford/AstraZeneca: 18 studies, Johnson & Johnson (J.J): 3 studies), 23 studies of mRNA vaccines (Pfizer-BioNTech: 17 studies and Moderna: 6 studies), and 4 studies of inactivated COVID vaccines (see Table 1).

**Table 1:** Gathering involved studies data and patient characteristics as well as adverse events

	Number of cases with adverse event	Percent %
Thrombotic thrombocytopenic purpura (TTP/VITT)	237	51.5%
Acute myocardial infarction (acute MI)	2	.4%
Myocarditis	22	5%
Idiopathic thrombocytopenic purpura (ITP)	4	.8%
Intracranial hemorrhage (ICH)	33	7%
Disseminated intravascular coagulopathy (DIC)	5	1%
Guillian-Barre Syndrome (GBS)	20	4%
Acute transverse myelitis (ATM)	9	2%
Acute disseminated poly radiculopathy (ADPR)	2	.4%
Miller Fisher syndrome (MFS)	1	.2%
Acute disseminated encephalomyelitis (ADEM)	2	.4%
Inflammatory myositis	3	.6%
Renal complications	41	9%
Erythema nodosum (EN)	1	.2%
Sarcoid reaction	1	.2%
Cutaneous vasculitis	1	.2%
Steven Johnson syndrome (SJS)	1	.2%
Uveitis	1	.2%

Twenty-three were case report studies, 7 were retrospective studies, 3 case series studies, and 1 cohort study as shown in Table 1.

In our research, 460 cases of adverse event post-vaccination reported, their age ranged from 15 to 75 with average of 49 years. Male to female ratio was 1.26:1.

Among 460 cases vaccinated for COVID (involving viral vector-based vaccines, mRNA vaccine, and inactivated virus vaccine), the following adverse events reported:

- ➣ Two hundred thirty-seven cases of thrombosis, ranged from cerebral venous thrombosis, thrombosis thrombocytopenic purpura, and pulmonary embolism [4–11]
- ➣ Two cases of acute myocardial infarction (AMI) [12]
- ➣ Twenty-two cases of myocarditis [13–15]
- ➣ Four cases of idiopathic thrombocytopenia (ITP) [16–19]
- ➣ Thirty-three cases of intracranial hemorrhage (ICH) [5]
- ➣ Five cases of disseminated intravascular coagulopathy (DIC) [10]
- ➣ Twenty cases of Guillian-Barre syndrome (GBS) [20–23]
  - ➣ Nine case of acute transverse myelitis (ATM) [24]
  - ➣ Two cases of acute demyelinating polyradiculopathy (ADPR) [25]
  - ➣ One case of Miller Fisher syndrome (MFS) [26]
  - ➣ Two cases of acute disseminated encephalomyelitis (ADEM) [27, 28]
  - ➣ Three inflammatory myositis [2]
  - ➣ Forty-one cases of renal adverse events (Ig A nephropathy, anti-GBM nephropathy, ANCA, and paucimmune glomerulopathy) [29, 30].
  - ➣ One case of erythema nodosum (EN) [31].
  - ➣ One case of sarcoid reaction [32].
  - ➣ One case of cutaneous vasculitis [1].
  - ➣ One case of Steven Johnson syndrome (SJS) [33]
  - ➣ One case of uveitis [34]
  - ➣ Seventy-four cases of other ocular adverse events (acute macular neuroretinopathy/facial palsy/choroiditis) [35]

Average time lapsed after vaccine in the above adverse events was 10–11 days.

Specific management of each adverse event ranges from steroids, IV immunoglobulin, plasma exchange, immunosuppressant, and topical steroids (eye/derma/mucous membrane).

**Fig. 2:**
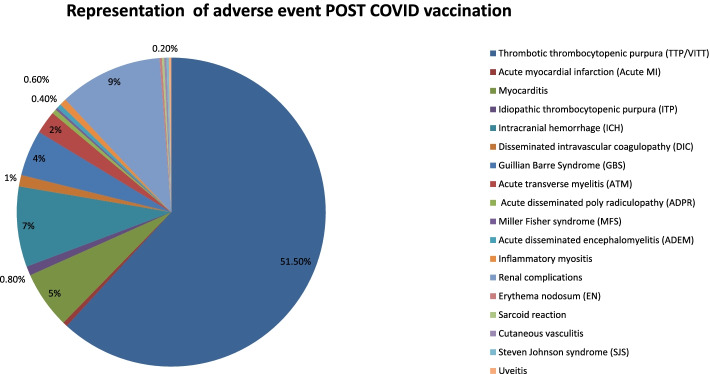
Different adverse events post-COVID vaccination in the study population in number and percentages

According to frequency of cases, the most prevalent adverse event was thrombosis/and related events as cerebral venous thrombosis, pulmonary embolism, and acute myocardial infarction. As well ocular and renal events are prevalent rather than neurological events (Fig. 2).

- ➣ Subgrouping of vaccinated cases according to used vaccine revealed three groups:
  - Group 1: Pts received viral vector-based vaccines with adverse events
  - Group 2: Pts received mRNA-based vaccines with adverse events
  - Group 3: Pts received inactivated virus vaccines with adverse events

### Comparing each two groups as regards adverse events done and revealed the following results

#### Comparing group 1 to group 2

Viral vector-based vaccine group (group 1) showed highly significant increase in cases of thrombotic thrombocytopenic purpura/cerebral venous thrombosis/pulmonary embolism with *p* < 0.0001

In the same context, group 1 had statistically significant increase in cases of DIC (*P* = 0.0316), GBS (*P* = 0.0036) and ATM (*P* = 0.0089). On the other hand, mRNA-based vaccine (group 2) showed highly significant increase in cases of renal complication, ocular complications and myocarditis (*p* < 0.0001).

Group 1 had highly significant increase in number of cases (314) compared to group 2 (142) (*p* < 0.0001).

See Table 2 for comparison between group 1 (pts received viral vector-based vaccines with adverse events) vs group 2 (pts received mRNA-based vaccines with adverse events).

**Table 2:** Comparison between group 1 (pts received viral vector-based vaccines with adverse events) vs group 2 (pts received mRNA-based vaccines with adverse events)

	Viral vector vaccine	Percentage	mRNA vaccines	Percentage	DIFF/95 CI/chi-squared/DF/significance
**Thrombosis/thrombocytopenia**	**236**	**51.30%**	**1**	**0.20%**	**51.1%/46.4340–55.6410%/313.779/1/*p*** **< 0.0001**
**ICH**	**33**	**7%**	**0**	**0**	**7%/4.8418–9.7052%/33.332/1/*p*** **< 0.0001**
**DIC**	**5**	**1%**	**0**	**0**	**1%/− 0.0148–2.3981%/4.618/1/*P*** **= 0.0316**
**GBS**	**16**	**4%**	**5**	**1%**	**3%/0.9911–5.2812%/8.483/1/*P*** **= 0.0036**
**Acute TM**	**7**	**2%**	**1**	**0.20%**	**1.8%/0.4357–3.5402%/6.842/1/*P*** **= .0089**
**ADPR (acute demyelinating polyradiculopathy)**	**2**	**0.40%**	**0**	**0**	**.4%/− 0.4793–1.5170%/1.842/1/*P*** **= 0.1748**
**ADEM: (acute disseminated encephalomyelitis)**	**1**	**0.20%**	**1**	**0.20%**	**0%/− 1.0055–1.0055%/0.000/1/*P*** **= 1.0000**
**Inflammatory myositis**	**3**	**0.60%**	**0**	**0**	**.6%/− 0.3215–1.8223%/2.765/1/*P*** **= 0.0963**
**Erythema nodosum**	**1**	**0.20%**	**0**	**0**	**.2%/− 0.6448–1.1916%/0.920/1/*P*** **= 0.3375**
**Renal complication**	**3**	**0.60%**	**36**	**8%**	**7.4%/4.9308%-10.2697%/30.573/1/*P*** **< 0.0001**
**Acute macular neuroretinopathy/facial palsy/choroiditis**	**7**	**2%**	**66**	**14%**	**12%/8.6447–15.5942%/44.951/1/*P*** **< 0.0001**
**Cutaneous vasculitis**	**0**	**0.00%**	**1**	**0.20%**	**.2%/− 0.6448–1.1916%/0.920/1/*P*** **= 0.3375**
**Myocarditis**	**0**	**0.00%**	**22**	**5%**	**5%/3.1579–7.3908%/23.564/1/*P*** **< 0.0001**
**Acute myocardial infarction**	**0**	**0.00%**	**2**	**0.40%**	**.4%/− 0.4793–1.5170%/1.842/1/*P*** **= 0.1748**
**ITP**	**0**	**0.00%**	**4**	**0.80%**	**.8%/− 0.1674–2.1148%/3.691/1/*P*** **= 0.0547**
**Miller Fisher S**	**0**	**0.00%**	**1**	**0.20%**	**.2%/− 0.6448–1.1916%/0.920/1/*P*** **= 0.3375**
**Sarcoid reaction**	**0**	**0.00%**	**1**	**0.20%**	**.2%/− 0.6448–1.1916%/0.920/1/*P*** **= 0.3375**
**Steven Johnson Syndrome**	**0**	**0.00%**	**1**	**0.20%**	**.2%/− 0.6448–1.1916%/0.920/1/*P*** **= 0.3375**
**Uveitis**	**0**	**0.00%**	**0**	**0.00%**	**0%/− 0.8282–0.8282%**

*ICH* intracranial hemorrhage, *DIC* disseminated intravascular coagulopathy, *GBS* Guillian-Barre Syndrome, *ATM* acute transverse myelitis, *ADPR* acute demyelinating polyradiculopathy, *ADEM* acute disseminated encephalomyelitis, *ITP* idiopathic thrombocytopenic purpura

#### Comparing group 2 to group 3

mRNA vaccine group (group 2) showed highly significant increase in cases of renal complication, ocular complications, and myocarditis (*p* < 0.0001), compared to inactivated virus-based vaccines group (group 3). Group 2 had significant increase in cases of GBS cases (*p* = 0.0316).

Highly significant increase in number of cases observed in group 2 (142) compared to group 3 [4] (*p* < 0.0001).

See Table 3 for comparison between group 2 (pts received mRNA-based vaccines with adverse events) vs group 3 (pts received inactivated virus-based vaccines with adverse events).

**Table 3:** Comparison between group 2 (pts received mRNA-based vaccines with adverse events) vs group 3 (pts received inactivated virus-based vaccines with adverse events)

	Group 2 (mRNA vaccines)	Percentage	Group 3 (inactivated virus vaccine)	Percentage	DIFF/95 CI/chi-squared/DF/significance
**Thrombosis/thrombocytopenia**	**1**	**0.20%**	**0**	**0%**	**.2%/− 0.6448–1.1916%/0.920/1/*P*** **= 0.3375**
**ICH**	**0**	**0**	**0**	**0%**	**0%/− 0.8282–0.8282%**
**DIC**	**0**	**0**	**0**	**0%**	**0%/− 0.8282–0.8282%**
**GBS**	**5**	**1%**	**0**	**0%**	**1%/− 0.0148–2.3981%/4.618/1/*P*** **= 0.0316**
**Acute TM**	**1**	**0.20%**	**1**	**0.20%**	**0%/− 1.0055–1.0055%/0/1/*P*** **= 1.0000**
**Acute demyelinating polyradiculopathy**	**0**	**0**	**0**	**0%**	**0%/− 0.8282–0.8282%**
**Acute disseminated encephalomyelitis**	**1**	**0.20%**	**0**	**0%**	**.2%/− 0.6448-1.1916%/0.920/1/*P*** **= 0.3375**
**Inflammatory myositis**	**0**	**0**	**0**	**0%**	**0%/− 0.8282–0.8282%**
**Erythema nodosum**	**0**	**0**	**0**	**0%**	**0%/− 0.8282–0.8282%**
**Renal complication**	**36**	**8%**	**1**	**0.20%**	**7.8%/5.4365–10.6460%/35.550/1/*P*** **< 0.0001**
**Acute macular neuroretinopathy/ facial palsy/ choroiditis**	**66**	**14%**	**1**	**0.20%**	**13.8%/10.7601–17.2739%/66.335/1/*P*** **< 0.0001**
**Cutaneous vasculitis**	**1**	**0.20%**	**0**	**0%**	**.2%/− 0.6448–1.1916%/0.920/1/*P*** **= 0.3375**
**Myocarditis**	**22**	**5%**	**0**	**0%**	**5%/3.1579%/7.3908%/23.564/1/*P*** **< 0.0001**
**Acute myocardial infarction**	**2**	**0.40%**	**0**	**0%**	**.4%/− 0.4793–1.5170%/1.842/1/*P*** **= 0.1748**
**ITP**	**4**	**0.80%**	**0**	**0%**	**.8%/− 0.1674–2.1148%/3.691/1/*P*** **= 0.0547**
**Miller Fisher S**	**1**	**0.20%**	**0**	**0%**	**.2%/− 0.6448–1.1916%/0.920/1/*P*** **= 0.3375**
**Sarcoid reaction**	**1**	**0.20%**	**0**	**0%**	**.2%/− 0.6448–1.1916%/0.920/1/*P*** **= 0.3375**
**Steven Johnson Syndrome**	**1**	**0.20%**	**0**	**0%**	**.2%/− 0.6448–1.1916%/0.920/1/*P*** **= 0.3375**
**Uveitis**	**0**	**0.00%**	**1**	**0.20%**	**.2%/− 0.6448–1.1916%/0.920/1/*P*** **= 0.3375**
	**142**	**31%**	**4**	**0.80%**	**30.2%/25.9377–34.5977%/156.703/1/*P*** **< 0.0001**

*ICH* intracranial hemorrhage, *DIC* disseminated intravascular coagulopathy, *GBS* Guillian Barre Syndrome, *ATM* acute transverse myelitis, *ADPR* acute demyelinating polyradiculopathy, *ADEM* acute disseminated encephalomyelitis, *ITP* idiopathic thrombocytopenic purpura

#### Comparing group 1 to group 3

Viral vector-based vaccine group (group 1) showed highly significant increase in cases of thrombotic thrombocytopenic purpura/cerebral venous thrombosis/pulmonary embolism with *p* < 0.0001

In the same context, group 1 had highly significant increase in cases of ICH (*P* = 0.0316), GBS (*p* < 0.0001). Significant increase (*p* < 0.05) in cases of DIC, ATM, and ocular complications was observed in group 1 compared to group 3.

Group 1 had highly significant increase in number of cases (314) compared to group 3 [4] (*p* < 0.0001).

See Table 4 for comparison between group 1 (pts received viral vector-based vaccines with adverse events) vs group 3 (pts received inactivated virus-based vaccines with adverse events).

**Table 4:** Comparison between group 1 (pts received viral vector-based vaccines with adverse events) vs group 3 (pts received inactivated virus-based vaccines with adverse events)

	Group 1 (viral vector vaccine)	Percentage	Group 3 (inactivated virus vaccine)	Percentage	DIFF/95 CI/chi-squared/DF/significance
**Thrombosis/thrombocytopenia**	**236**	**51.30%**	**0**	**0%**	**51.3%/46.6659–55.8379%/317.046/1/*p*** **< 0.0001**
**ICH**	**33**	**7%**	**0**	**0%**	**7%/4.8418–9.7052%/33.332/1/*p*** **< 0.0001**
**DIC**	**5**	**1%**	**0**	**0%**	**1%/− 0.0148–2.3981%/4.618/1/*p*** **= 0.0316**
**GBS**	**16**	**4%**	**0**	**0%**	**4%/2.3366–6.2045%/18.755/1//*p*** **< 0.0001**
**Acute TM**	**7**	**2%**	**1**	**0.20%**	**1.8%/0.4357–3.5402%/6.842/1/*p*** **= 0.0089**
**Acute demyelinating polyradiculopathy**	**2**	**0.40%**	**0**	**0%**	**.4%/− 0.4793–1.5170%/1.842/1/*p*** **= 0.1748**
**Acute disseminated encephalomyelitis**	**1**	**0.20%**	**0**	**0%**	**.2%/−0.6448–1.1916%/0.920/1/*p*** **= 0.3375**
**Inflammatory myositis**	**3**	**0.60%**	**0**	**0%**	**.6%/− 0.3215–1.8223%/2.765/1/*p*** **= 0.0963**
**Erythema nodosum**	**1**	**0.20%**	**0**	**0%**	**0.2%/− 0.6448–1.1916%/0.920/1/*p*** **= 0.3375**
**Renal complication**	**3**	**0.60%**	**1**	**0.20%**	**.4%/− 0.6708–1.6336%/0.923/1/*p*** **= 0.3368**
**Acute macular neuroretinopathy/facial palsy/choroiditis**	**7**	**2%**	**1**	**0.20%**	**1.8%/0.4357–3.5402%/6.842/1/*p*** **= 0.0089**
**Cutaneous vasculitis**	**0**	**0.00%**	**0**	**0%**	**0%/− 0.8282–0.8282%**
**Myocarditis**	**0**	**0.00%**	**0**	**0%**	**0%/− 0.8282–0.8282%**
**Acute myocardial infarction**	**0**	**0.00%**	**0**	**0%**	**0%/− 0.8282–0.8282%**
**ITP**	**0**	**0.00%**	**0**	**0%**	**0%/− 0.8282–0.8282%**
**Miller Fisher S**	**0**	**0.00%**	**0**	**0%**	**0%/− 0.8282–0.8282%**
**Sarcoid reaction**	**0**	**0.00%**	**0**	**0%**	**0%/− 0.8282–0.8282%**
**Steven Johnson Syndrome**	**0**	**0.00%**	**0**	**0%**	**0%/− 0.8282–0.8282%**
**Uveitis**	**0**	**0.00%**	**1**	**0.20%**	**.2%/− 0.6448–1.1916%/0.920/1/*P*** **= 0.3375**

*ICH* intracranial hemorrhage, *DIC* disseminated intravascular coagulopathy, *GBS* Guillian Barre Syndrome, *ATM* acute transverse myelitis, *ADPR* acute demyelinating polyradiculopathy, *ADEM* acute disseminated encephalomyelitis, *ITP* idiopathic thrombocytopenic purpura

Among the included studies, 8 (AZA), 4 (Moderna), 10 (Pfizer), and 1 (inactivated) studies mentioned comorbidities in vaccinated individuals. Twenty studies, of which 19 were case reports/case series vs 1 retrospective study.

See Table 5 for gathering studies that mentioned comorbidities in vaccinated individuals.

**Table 5:** Studies that showed comorbidities of involved vaccinated patients

Author	Name of vaccine	Type of study	Comorbidity	Adverse event
Ng XL et al. 2021 [35]	66 Pfizer-BioNTech /7 Oxford/AstraZeneca /1BBIBP Corv	Retrospective (observational study)	Diabetes, rheumatolgical disorders	Acute macular neuroretinopathy/facial palsy
Hal F et al. 2021 [31]	Oxford/AstraZeneca	Case report	Cancer breast	Erythema nodosum
Biswas A et al. 2021 [21]	Oxford/AstraZeneca	Case report	Non-syndromic retinitis pigmentosa	Guillian Barre syndrome (GBS)
Boonyawat K et al. 2022 [8]	Oxford/AstraZeneca	Case reports	Diabetes, hypertension, and hyperlipidemia	Thrombotic thrombocytopenic purpura (TTP)
Nasuelli NA et al. 2021 [25]	Oxford/AstraZeneca	Case report	Hypertension and hyperuricemia	Acute demyelinating polyradiculopathy
Takata J et al. 2021 [27]	Oxford/AstraZeneca	Case report	Non-syndromic retinitis pigmentosa	Acute disseminated encephalomyelitis
Greinacher A et al. 2021 [10]	Oxford/AstraZeneca	Case series	Von Willebrand disease, anti-cardiolipin antibodies, and factor V Leiden	Thrombotic thrombocytopenic purpura (TTP)/ disseminated intravascular coagulopathy (DIC)
Hasan T et al. 2021 [22]	Oxford/AstraZeneca	Case report	Bronchiectasis, asthma, osteoporosis and migraine.	Guillian Barre syndrome (GBS)
Klomjit N et al. 2021 [29]	6 Pfizer-BioNTech/7 Moderna		Cancer/diabetes/autoimmune	Renal complications
Akinosoglou K et al. 2021 [1]	Pfizer-BioNTech	Case report	Cutaneous small cell vasculitis	Cutaneous vasculitis
Fueyo-Rodriguez O et al. 2021 [16]	Pfizer-BioNTech	Case report	Hypo thyroidism, hypertension and pre-diabetes	Idiopathic thrombocytopenic purpura (ITP)
Vogrig A et al. 2021 [28]	Pfizer-BioNTech	Case report	History of post-infectious rhombencephalitis	Acute disseminated encephalomyelitis
Innao V et al. 2022 [11]	Pfizer-BioNTech	Case reports	Nodular sclerosis classical Hodgkin lymphoma (NScHL), in stage VI B disease	Thrombotic thrombocytopenic purpura (TTP)
Bouattour N et al. 2022 [23]	Pfizer-BioNTech	Case report	Type II diabetes	Guillian Barre Syndrome
Malayala SV et al. 2021 [17]	Pfizer-BioNTech	Case report	Rheumatoid arthritis, scleroderma, mixed connective tissue disease, hypertension, cardiac disease, and osteopenia	Idiopathic thrombocytopenic purpura (ITP)
Jasaraj RB et al. 2021 [18]	Pfizer-BioNTech	Case report	Hypertension, type 2 diabetes mellitus, hypothyroidism, depression, vitamin B12 deficiency,	Idiopathic thrombocytopenic purpura (ITP)
Nishiguchi Y et al. 2021 [26]	Pfizer-BioNTech	Case report	Diabetes mellitus and diabetic ophthalmoplegia	Miller Fisher syndrome
Helms JM et al. 2021 [19]	Moderna	Case report	Hypertension, gout, hyperlipidemia, and non-ischemic cardiomyopathy	Idiopathic thrombocytopenic purpura (ITP)
Sekar A et al. 2021 [30]	Moderna	Case report	Hypertension	Renal complication
Sung JG et al.2021 [12]	Moderna	Case reports	Hypertension, hyperlipidemia, diabetes mellitus, coronary artery disease	Acute myocardial infarction

## Discussion

### Review in context

Reviewed published literature includes case reports, series, and narrative studies. They all recorded cases of adverse events post-COVID vaccination. Each study randomly reported adverse events related to one type of vaccine on retrospective base as case report vs series. Narrative reviewing as well as spot light on adverse events in context of need for further studies. While in the era of progressive mass vaccination, analyzing such data to decrease morbidity and mortality post-vaccination is essential in facing epidemic.

### Added value of this study and implications

Analysis of available data favors differential risk profiling of the available vaccines so that we can subsequently select appropriately the target population with utmost benefit and least harm per each vaccine. Further research is needed in the same context on larger scale, as well as studying future panels used in stratifying population in relation to suitability to which vaccine against COVID-19.

Basically, vaccinations are used to decrease the burden of infection to boost vaccine efficacy, and adjuvants are often added to stimulate immune systems. However, such adjuvants can lead to autoimmune or inflammatory syndrome [35]. Vaccine-induced adverse events had been established with many vaccines. COVID-19 vaccines induced adverse events as well.

Among mechanisms involved in acute autoimmune response post-vaccination is molecular mimicry between host antigens and spike proteins [1].

There are lots of reports on adverse events post-COVID vaccination. Cases related to all available vaccines are registered from all over the world.·

In our study, 237 case of post-vaccination thrombosis/related thrombotic presentations were recorded. It was the dominant adverse event. There was significant increase in viral vector vaccines group (AstraZeneca/JJ) compared to other groups.

In a study by Favaloro E., [6], cases of suspected vaccine induced thrombotic thrombocytopenia (VITT) 4–16 days post-AstraZeneca vaccine were reported.

In the same vein, Markus [4]. highlighted recent reports of coagulopathy associated with COVID-19 vaccination particularly the AstraZeneca COVID-19 vaccine 12 days (median) after vaccine. According to Perry et al. [7], vaccine-induced immune thrombotic thrombocytopenia (VITT) manifested as cerebral venous thrombosis after first dose of AstraZeneca.

In agreement, another review done by García-Azorín, D., et al. [5] identified 77 cases of cerebral venous thrombosis post-vaccination 8 days (median) after vaccination.

On the other hand; there are case reports in which two mRNA vaccines, mRNA-1273 (Moderna), and bnt162b2 (Pfizer–BioNTech), are associated with thrombocytopenia, purpura, and mucosal bleeding rather than thrombosis [7]. In agreement, at least 25 reports of “immune thrombocytopenia” (ITP) or “thrombocytopenia” following the Moderna or Pfizer vaccine were added to the vaccine adverse event reporting system (VAERS) in the USA [19].

A case of ITP reported by Fueyo-Rodriguez, O., et al. [16], 12 h after mRNA COVID-19 vaccine BNT162b2. The case was treated accordingly with immunoglobulin and steroids. Another case of refractory thrombocytopenia 1 day after MODERNA SARS-COV-2 vaccine was recorded by Helms et al. [19]. The mechanism of post-vaccination thrombocytopenia is presumed to be immune-mediated and related to hyper function of B cells observed in ITP.

Reffering to potential neurological complications post-COVID-19 vaccines, many reviews are available worldwide [24]. Cases of GBS and ATM recorded with significant increase in viral vector vaccine group compared to other two groups, as well as mRNA vaccine group showed significant more cases of GBS compared to group 3.

In a review of nine articles, 18 patients with COVID vaccine-induced Guillian-Barre (SCOVaG) were reported by Finsterer J. et al. [20], 3 h to 39 days after the first dose of the vaccine. The Astra Zeneca (ASZ) vaccine was used in 14 patients, the Pfizer vaccine in 3 patients, and the Johnson & Johnson vaccine was used in 1 patient [20].

During Astra Zeneca vaccine (AZD1222) clinical trials among 11,636 participants, three cases of post-vaccination acute transverse myelitis (ATM) observed [24]. Vogrig et al. [28] reviewed a case of acute disseminated encephalomyelitis (ADEM) 2 weeks after the first dose of the Pfizer-BioNTech COVID-19 vaccine (Comirnaty).

In relation to the musculoskeletal system, Maramattom et al. [2] suggested that inflammatory myositis could develop secondary to COVID-19-related immune disorder.

Renal complications were significantly more prevalent in mRNA vaccine group, compared to other two groups according to our work.

Hakroush S and Tampe B [36] present the first case of ANCA-associated vasculitis presenting with massive rhabdomyolysis and pauci-immune crescentic GN after Pfizer-BioNTech COVID-19 (mRNA vaccine) [36]. In another review, five cases of pauci-immune crescentic ANCA GN reported after the second dose of COVID-19 mRNA vaccination [36].

In the same vein, a case of anti-neutrophil cytoplasmic antibody (ANCA) glomerulonephritis 2 weeks after receiving the COVID-19 (MODERNA) vaccine was reported by Sekar et al. [30]. In a case series of 13 patients post-mRNA vaccines, newly diagnosed/flares of GN were attributed to new IgA nephropathy, membranous nephropathy, primary FSGS, and atypical anti-GBM nephritis [29].

Dermatologically, Hali, et al. [31] discussed a case of erythema nodosum (EN) occurring 48 h after second dose of “AstraZeneca”. Another dermatological adverse event is Stevens-Johnson syndrome (SJS). Elboraey, MO., and Essac EF., discussed a case of (SJS) that occurred after the second dose of the Pfizer COVID-19 vaccine [33].

Moving to ocular adverse events in relation to COVID vaccines, our study revealed significant increase in ocular adverse events in mRNA vaccine group compared to other groups. However, ocular complications were reported with all groups. In same context, Ng XL, et al. [35] confirmed ocular complications were reported after mRNA- and vector-labored vaccines. Complications included facial nerve palsy/Bell’s palsy, abducens nerve palsy, AMN, superior ophthalmic vein (SOV) thrombosis, and uveitis.

Pan et al. [34] reported a case of bilateral posterior uveitis post-vaccination with inactivated COVID-19 vaccine.

As regards myocarditis as adverse event, it was significantly reported more with mRNA vaccine group compared to other two groups.

Twenty-two cases of myocarditis were reported by Parra-Lucares, et al. [13], Dionne et al. [14], and Abu Mouch et al. [15] after mRNA vaccines.

According to studies mentioned, the following are comorbidities in vaccinated individuals:

- ✓ Diabetes, rheumatological disorders, cancer breast, non-syndromic retinitis pigmentosa, hypertension and hyperlipidemia, hyperuricemia, von Willebrand disease, anti-cardiolipin antibodies, factor V Leiden, bronchiectasis, asthma, and osteoporosis and migraine were mentioned in AstraZeneca-vaccinated individuals
- ✓ On the other hand, in Pfizer-vaccinated individuals report cancer, diabetes, cutaneous small cell vasculitis, hypo thyroidism, pre-diabetes, post-infectious rhombencephalitis, nodular sclerosis classical Hodgkin lymphoma, rheumatoid arthritis, scleroderma, mixed connective tissue disease, hypertension, cardiac disease, osteopenia, depression, and vitamin B12 deficiency were reported as comorbidities.
- ✓ In Moderna-vaccinated individuals: hypertension, gout, hyperlipidemia and non-ischemic cardiomyopathy, diabetes mellitus, coronary artery disease, cancer, and autoimmune disease were recorded in post-vaccinated individuals as comorbidities.
- ✓ One study reported diabetes and rheumatological disorders as comorbidities in vaccinated individuals with inactivated viral vaccine.

Nineteen out of 20 studies were in form of case reports vs case series. So, evaluation of relationship between comorbidities and adverse events need further researches in the same context on larger scales.

In our study, there were limitations to the data provided.

- ✓ There are 4 studies done on the inactivated vaccine, in comparison to 21 study on viral vector-based vaccines and 23 study of mRNA vaccines; this makes the comparison to inactivated vaccine inaccurate. It is all about limited published work on inactivated vaccines compared to viral vector and mRNA vaccines.
- ✓ Another limitation of available data is due to under recording and registry in many countries. So, to judge on the adverse effects of inactivated COVID vaccines, we need larger scale studies in the same context.
- ✓ As well case reports and series were involved in our study due to rarity of some adverse events and under reporting besides
- ✓ Low awareness and suspicion of physicians as well as patients. So, it is imperative that physicians, other medical staff, and vaccinated persons be sensitized to the need of reporting any side effect of the vaccine given for COVID-19.

Based on the available data, risk profiling of vaccine/patient in each case may be possible soon.

We observed viral vector vaccines (especially with younger than 50 years) causes thrombosis/and thrombosis-related complications as well as neurological adverse events. However, mRNA vaccines causes more ocular, renal, and myocarditis (especially in younger patients).

- ✓ The role of health authorities in follow up and registration of any adverse effects from the given vaccines need to be activated. This will help build a good data base and direct the future research on the development of safe efficacious vaccine

Also, a dedicated international registry for recording adverse effects post-COVID-19 vaccination worldwide. It could facilitate international work plan to accurately profile different vaccine per individual patient. And hence decrease morbidity and mortality post-vaccination.

## Conclusions

No doubt, emergence of various COVID vaccines had limited morbidity and mortality of COVID global pandemic.

A hyper-reactive or prolonged reactogenicity against host antigens can lead to more severe adverse events.

The growing need for screening systems of post-vaccine adverse events should be acted in prospective and retrospective manner to avoid under recording of cases.

Various systems affected post-COVID vaccines inoculation, supporting the notion of immune/inflammatory nature of adverse event. Such adverse events as neurological, ocular, dermatological, hematological, cardiac, and renal.

During the current period of COVID-19 vaccination, a high index of suspicion is required to identify thrombotic/neurological adverse events following vaccination especially viral vector-labored vaccines, and renal/ocular/myocarditis following vaccination especially mRNA vaccines.

It is worth noting that the ChAdOx1 nCoV-19 vaccine triggers immune response by nCoV-19 spike protein, whereas the mRNA-vaccine induces antibody response with a lipid-nanoparticle-encapsulated mRNA. This may explain differential adverse profile of each vaccine.

It is important as well to initiate preventive, screening/surveillance system, and management for post-COVID vaccination adverse events.

Particular association between individual vaccine and specified adverse event (e.g., AstraZeneca/thrombophilia) must be revised by generating company and judged by WHO to avoid adverse event, mortality, and morbidity.

Vaccine-specific {differential} adverse effect in relation to reported adverse events to specific vaccine, the following were observed:

1. The most famous association is AstraZeneca/thrombophilia. Such association reported more in middle-aged male.
2. AstraZeneca/EN (erythema nodosum)
3. mRNA vaccines/glomerulopathy/myocarditis
4. mRNA vaccines/ITP (idiopathic thrombocytopenia)
5. Pfizer/SJS (Steven Johnson Syndrome)
6. Inactivated vaccines/ocular adverse events.
7. Identification of high-risk patients susceptible to specific adverse event post-vaccine is essential.

Risk stratification of individuals have to guide subsequent selectivity of safe efficacious vaccine based on vaccine/individual risk profile.

It remains an open question, when these phenomena do occur whether a second dose should be administered.

More data is needed to assess such association on larger scale.

## Data Availability

Better to be open access.
